# Sustaining social license: nuclear weapons and the art of legitimation

**DOI:** 10.1057/s41311-022-00404-w

**Published:** 2022-09-09

**Authors:** Kjølv Egeland

**Affiliations:** Sciences Po, Paris, France

**Keywords:** Legitimation, Public relations, Institutional entrenchment, Strategic narratives, Propaganda, Hegemony-building

## Abstract

Several observers have in recent years discussed the prospects for advancing arms control and disarmament through determined efforts at delegitimizing nuclear weapons. But surprisingly little attention has been devoted to the question of how nuclear weapons are legitimized in the first place. And while there is widespread agreement that struggles for legitimacy make up a pervasive feature of political life generally and nuclear politics specifically, available typologies of legitimation invariably comprise only a subset of the varied ways in which actors struggle to build or retain a social license to operate. Drawing on scholarship on propaganda, marketing, and legitimacy-seeking, this article offers an investigation of the full range of actions nuclear-armed governments and defence contractors undertake to sustain acceptance for the continued development and deployment of nuclear arms. Four broad categories of legitimation are identified: discursive legitimation, institutional legitimation, behavioural legitimation, and legitimation through information control.

## Introduction

Why does the Russian military have Orthodox priests sprinkle holy water on Russia’s intercontinental ballistic missiles? Why did the British Ministry of Defence stop publishing the official safety ratings for its Trident nuclear weapon system? And why does Lawrence Livermore, one of the laboratories that produce and maintain nuclear warheads for the US government, sponsor the Girl Scouts of America? The answers to these questions may seem obvious; in each case, the actor in question is at least in part acting to bolster, or pre-empt the possible loss of, its social license to operate or maintain a policy. But the activities in question all fall outside standard typologies of political legitimation. Investigating the ways in which pro-nuclear actors act to legitimate the development and deployment of nuclear arms, this article proposes an expanded taxonomy of legitimation that, in focusing on what legitimacy-seekers do in practice, goes beyond direct, self-legitimating rhetoric to capture a broader array of actions aimed at enhancing or sustaining social license.

The nuclear weapons enterprise has long been subjected to normative condemnation and political contestation (Ritchie 2013; Wittner 1993–2003). Use of nuclear weapons, on any appreciable scale, would have devastating humanitarian and environmental consequences. In the words of the international legal scholar Ian Brownlie (1965: 451), preparing for the use of nuclear weapons ‘certainly involves the preparation of crimes against humanity’. According to the International Court of Justice (ICJ), the use of nuclear arms would generally be ‘contrary to the rules of international law applicable in armed conflict’ (ICJ 1996: 44). The production and maintenance of nuclear weapons have also been argued to steal resources from urgent political imperatives such as decarbonisation and poverty alleviation (Perry 2021; Pope Francis 2019). The goal of nuclear disarmament accordingly enjoys strong popular support across the world (Egeland and Pelopidas 2021: 249; Kafura 2020; International Committee of the Red Cross 2020). Yet nuclear arms are still around. In fact, all nine of the world’s nuclear-armed states (China, France, India, Israel, North Korea, Pakistan, Russia, the United Kingdom, and the United States) are currently engaged in long-term projects to expand or rebuild their nuclear capabilities (Kristensen and Korda 2021). How do those responsible build social license for these policies? That is the question this article seeks to answer.

Several scholars have written insightfully about the nature and role of legitimacy in international relations and global governance; there is broad acceptance that legitimacy is a prized asset among international actors and that legitimation plays an important role in fostering compliance with international institutions and rules (Hurd 1999; Bernstein 2004; Zürn 2004; Clark 2005; Buchanan and Keohane 2006; Collingwood 2006; Scholte 2011; Keohane 2011; Tallberg and Zürn 2019). Less has been written about the ways in which actors struggle for legitimacy in practice (Bäckstrand and Söderbaum 2018). However, mapping out what, precisely, states and organizations do to build or maintain legitimacy is important not only to make sense of a myriad of corporate, domestic, and foreign policies, but also as a step towards a richer understanding of institutional stability, contestation, and change. Many contemporary political and social movements seek change through efforts at ‘stigmatizing’, ‘abolishing’, or otherwise dismantling existing institutions and practices, be it the continued extraction of fossil fuels, the development and deployment of indiscriminate weapons, or global governance itself (Princen et al. 2015; Garcia 2015; Bäckstrand and Söderbaum 2018). Understanding how contested institutions and policies are legitimated and ensconced from pressure is essential to grasping the ways in which those same institutions and policies are perpetuated and, potentially, vulnerable to abolitionist pressures (Grigorescu 2015). This article is thus aimed at contributing to ongoing debates on legitimation (Dajani et al. 2021; Gronau and Schmidtke 2016; Bäckstrand and Söderbaum 2018; Tallberg and Zürn 2019; Bexell et al. 2021; Sundaram 2021) and normative change in the global nuclear order and international society more broadly (Ritchie 2013, 2019; Biswas 2014; Sauer and Reveraert 2018; Considine 2019; Burke and Fishel 2020; Pelopidas 2021, 2022).

## Legitimation in theory and practice

The concept of legitimacy is often used in a narrow sense to refer to ‘beliefs within a given constituency or other relevant audience that a political institution’s exercise of authority is appropriate’ (Tallberg and Zürn 2019: 585). In this paper, drawing on Clark (2005), legitimacy is understood in a broader sense to refer to the general state or quality of being perceived or treated as socially acceptable, a trait that may attach not only to the exercise of authority by political institutions, but also to policies and behaviours enacted by individuals, companies, or movements. Legitimacy, in this view, amounts to ‘social acceptance’ (Zürn 2004: 260) or ‘social license’ (Cashore 2002: 513; Blanton and Blanton 2009). Thus conceived, legitimacy may be understood as a prerequisite for the perpetuation of contested policies, industries, and institutions. Along these lines, what I in this article refer to as practices of legitimation are understood as ‘socially meaningful patterns of action’ geared towards producing or maintaining social license (see Adler and Pouliot 2011: 17).

While those involved in struggles for legitimacy often act on the basis of carefully deliberated strategic objectives, ‘practice’ as understood here does not rely on assumptions of conscious, rule-following action. As one set of scholars point out, it is possible to study something as a meaningful pattern of action irrespective of whether those involved in that pattern consciously see themselves as acting out a particular practice or strategy (Andersen and Neumann 2012: 468). Like other practices, legitimacy-seeking often does not derive from conscious deliberation or strategizing but is frequently a product of ‘inarticulate, practical knowledge that makes what is to be done appear “self-evident” or commonsensical’ (Pouliot 2008: 258). Practices of legitimation can be distinguished from other social practices in that they have a clear role in what Considine (2019) calls ‘contests of legitimacy and value’, that is, the politics of producing or revoking social license to operate or maintain a policy. Following Bäckstrand and Söderbaum (2018), this paper proceeds from the understanding that legitimation and delegitimation are inextricably intertwined and must be analysed within the same framework. After all, any effort at legitimating a given actor or policy usually involves delegitimizing opposing views. Likewise, any effort at branding something as morally or politically unacceptable necessarily implies an implicit or explicit case in favour of an alternative.

Practices of legitimation are pervasive in both international and domestic politics. In Clark’s words (2005: 2–3), ‘actors within international society are engaged in endless strategies of legitimation, in order to present certain activities or actions as legitimate.’ After all, actors or institutions in command of legitimacy typically benefit in three key ways (Reus-Smit 2007: 163–64; Dowling and Pfeffer 1975: 126). First, they profit from active support and enthusiasm, for example by attracting interest from investors, visitors, or the best possible job applicants in recruitment processes. Second, policies and institutions that enjoy legitimacy typically benefit from compliance and acquiescence, allowing operation with minimal interference or regulation. Third, all else equal, actors that possess legitimacy benefit from lower levels of opposition, which in turn reduces the costs of compensating for lacking legitimacy with material coercion or bribery. Legitimacy, in this view, comes in degrees; an actor, policy, or institution can enjoy more or less legitimacy, translating into varying levels of access to the benefits described above. That said, as Reus-Smit argues (2007), an acute depletion of social acceptance may lead to a ‘crisis of legitimacy’, obliging the affected actor or institution to completely recalibrate the social bases of its legitimacy or face collapse or disempowerment. This article is concerned with socially meaningful patterns of action geared towards forestalling or preventing such crises of legitimacy from occurring, to wit, with efforts at defending a policy or institution that is contested but not yet suffering an acute crisis.

What I conceptualize here as practices of legitimation differ from traditional, profit-motivated marketing in that the legitimacy-seeker is struggling not only to increase its power or revenue but to build or retain its social license to operate. History suggests that successful legitimation can often make up the difference between institutional flourishing and failure; fur farming, production of cluster munitions, and slave trade provide examples of industries/policies that have lost legitimacy in many or most societies, resulting in legal prohibitions and market breakdown. Below, I discuss efforts by nuclear-armed governments and arms contractors at legitimating the continued development and deployment of nuclear weapons in the face of normative censure by civil society organizations, media actors, non-nuclear-weapon states, and many religious institutions.

Numerous analysts have identified specific practices and strategies of legitimation. For example, Ikenberry (2001: chapter 4) highlights how Britain and Russia deployed a strategy of ‘institutionalization’ to legitimate the European balance of power after the Napoleonic war; Crilley and Pears (2021) show how the CIA uses humour on Twitter to legitimate its actions; Devers et al. (2009: 264–65) unpack the politics of stigma and legitimacy ‘by association’; Goddard (2009) shows how Prussia legitimated its rise in the nineteenth century by deploying a series of narratives carefully designed to neutralize those likely to balance against it; Clark (2005: 203) examines the practice of rhetorical ‘reframing’ carried out by the US government to legitimate the invasion of Iraq in 2003; and Dingwerth et al. (2020) discuss the projection by international organizations of self-legitimating ‘democracy narratives’ aimed at boosting their standing. Human rights organizations have warned that repressive regimes in ‘need of sources of legitimacy’ are investing in sports teams and events to boost their popularity (Delaney 2020). Finally, there is widespread agreement that propaganda and censorship provide authoritarian leaders with powerful tools of legitimation (Gläßel and Paula 2020: 683).

However, no comprehensive typology of the full array of legitimation practices discussed in this article has been offered. Most existing typologies of legitimation focus only on discourse or styles of argumentation (Sundaram 2021; MacKay 2019: 719; Abulof 2015: 79; Vaara 2014; Reyes 2011). Others are tailored to the important, but narrower question, of how global governance institutions legitimate their exercise of political authority in international affairs (Grigorescu 2015; Gronau and Schmidtke 2016; Dingwerth et al. 2019; Tallberg and Zürn 2019; Dellmuth and Schlipphak 2020; Bexell et al. 2021). Most existing studies foreground good-faith interventions in political discourse, neglecting the darker arts of silencing dissenting voices, co-opting challengers, or building legitimacy through associations with third parties. While some might contend that such behaviours should not be understood as instances of legitimation, I suggest that all actions geared towards gaining or sustaining social license should be included in a unified framework.

The next sections lay out four broad types of legitimation. The first three are derived from Bäckstrand and Söderbaum’s (2018) distinction between ‘discursive’, ‘institutional’, and ‘behavioural’ practices of (de)legitimation. The fourth, introduced here as an addition to the Bäckstrand and Söderbaum typology, is adapted from Garth Jowett and Victoria O’Donnell’s theory of propaganda and concerns the control of information flows (Jowett and O’Donnell 2012). I argue that the control of information constitutes an important asset in contests of legitimacy and value and that efforts at suppressing or amplifying particular pieces of information should be understood as a form of legitimation along with discursive, institutional, and behavioural legitimacy-seeking.

## Legitimizing nuclear weapons: four categories of legitimation

### Discursive legitimation

Efforts at legitimation often take the form of speech acts, with those involved seeking to convince their audience that a given policy or institution is good, necessary, or at least not as bad as the alternative. The actors that sustain contested industries such as fossil fuel extraction, nuclear weapons production, and fur farming frequently engage in public outreach and debate to defend and justify the industry in question. In the words of Bäckstrand and Söderbaum (2018: 108), ‘discursive legitimation’ concerns the ‘use of argument and reason’ to establish legitimacy. In practice, legitimating agents often attempt to project ‘strategic narratives’ that allow them ‘to construct a shared meaning of the past, present and future of international relations in order to shape the opinions and behaviour of actors at home and overseas’ (Miskimmon et al. 2013: 176). For example, since the early 1990s, pro-nuclear actors within the US executive have told a compelling story about how the retention of a large nuclear arsenal provides an essential ‘hedge’ against hypothetical future dangers (Taylor 2010). With the Soviet Union gone – and with it the primary argument for a large US nuclear arsenal – a new justification for the American nuclear triad was required. In practice, the projection of this narrative amounted to the development of an ‘imagined future’ with constitutive effects on the present (see Pelopidas 2016, 2021).

Those invested in the nuclear enterprise typically alternate between promoting ‘positive’ arguments about the alleged necessity of nuclear deterrence and ‘negative’ arguments about the supposed perversity, jeopardy, or futility of disarmament (see Hirschman 1991; see also Pelopidas 2015). Discursive legitimation comprises a wide repertoire of sub-practices and tactics, including story-telling, conceptual reframing, and appeals to logic, evidence, emotions, or values (Suddaby and Greenwood 2005). In Russia, nuclear weapons are routinely portrayed by the government and government-aligned institutions as a vital, even divine, bulwark against alleged Western imperialism and efforts at subversion (Adamsky 2019). Russian President Vladimir Putin often appeals to emotive imaginaries that short-circuit Russian nationalism and pride with nuclear weapons. In 2018, for instance, in a speech announcing the procurement of a suite of new nuclear weapon systems, he suggested that the development of new nuclear capabilities was a means of counteracting purported humiliations inflicted on Russia. ‘They [the West] kept ignoring us, so listen to us now!’ (CBC News 2018).

Already in the 1980s, critical scholars developed the concept of ‘nukespeak’ to refer to the use by pro-nuclear actors of metaphors and euphemisms to portray nuclear weapons in a positive or neutral way (Chilton 1985; Cohn 1987). According to Edward Schiappa (1989: 255), nukespeak invariably instantiates one of two rhetorical strategies. The first of these is ‘domestification’, which refers to the normalization of nuclear arms through sanitizing metaphors and everyday language. For example, pro-nuclear actors frequently represent any development or manufacture of nuclear weapons simply as ‘modernization’. As another example, collective, transnational commitments to nuclear defence are typically referred to as ‘nuclear umbrellas’. Schiappa’s second mode of nukespeak, ‘bureaucratization’, describes the use of acronyms and technical terms that ensconce the nuclear domain from meaningful public oversight. For instance, the nuclear missile submarines currently under development in France are commonly referred to as ‘SNLE 3G’. Similarly, the US government’s ambition to rebuild the nuclear missile complex in the Midwest is ‘the GBSD program’. Another rhetorical mechanism is that of ‘reversification’, whereby a concept is given the opposite of its everyday or original meaning. For example, in European security debate, nuclear weapons and the practice of deterrence are often argued to provide ‘strategic autonomy’ despite the practice of nuclear deterrence being widely acknowledged to rest on the very opposite of autonomy, namely an embrace of mutual vulnerability (Egeland and Pelopidas 2021: 250). In the words of Laura Considine (2020: 55), nuclear language ‘does more than simply provide a neutral vehicle to describe the means and potential for nuclear disarmament: it also shapes our common understanding of what nuclear disarmament is, what it requires, and what its possibilities are.’ By extension, language is simultaneously an object and subject of the politics of legitimacy in global nuclear politics.

In contemporary nuclear affairs, discursive legitimation is pursued in numerous ways and on a myriad of platforms, involving everything from speechmaking and incantation to the publication of written material, contributions to traditional print media, participation in broadcast media, and outreach through social media. As an example of the latter, the US combatant command responsible for US ‘strategic deterrence’ (STRATCOM) maintains an active presence on Twitter, regularly disseminating colourful infographics and rebuttals of arguments for arms control and disarmament under the hashtag #MythMondays. According to one analyst, STRATCOM’s increasing presence on social media is a response to mounting online criticism of nuclear armament and purportedly wasteful defence spending (Foley 2020). Nuclear-armed states have also used more traditional advertising for discursive legitimation. For instance, on 17 December 2019, the Russian Armed Forces marked the Russian ‘strategic missile forces day’ by posting large billboards in Moscow depicting an intercontinental ballistic missile under the slogan ‘after us—silence’—an apparent allusion to the argument that, in the absence of an effective Russian nuclear arsenal, Russia would be wiped out by enemies (Novaya Gazeta 2019).^1^

### Institutional legitimation

Legitimation may also be pursued through the creation and manipulation of institutions, rules, and processes. As conceptualized by Bäckstrand and Söderbaum (2018: 108), ‘institutional legitimation’ describes the pursuit of legitimating agents of social acceptance through legalization, reforms, regulation, strategic norm creation, or institutional entrenchment. As an example of the latter, legitimating agents frequently endeavour to pulverize political resistance by co-opting existing or potential critics into status quo-oriented institutions (see Kruck and Zangl 2020). Such legitimation has been common within the nuclear non-proliferation regime. According to one scholar, critics of the prevailing nuclear order have been co-opted into multilateral institutions, at the United Nations and elsewhere, that provide ‘opportunities to vent spleen, rather than achieving much in terms of tangible results on either non-proliferation or disarmament’ (Ruzicka 2018: 379). A case in point is the Conference on Disarmament in Geneva, a standing negotiating forum comprised of delegations from 65 states. While in theory the Conference is supposed to advance nuclear disarmament, in practice its strict rules of procedure have allowed states that are unwilling to put their abolitionist rhetoric into action to quietly block the adoption of even a programme of work every year since the mid-1990s (Meyer 2021). As another example, in the mid-2000s, the United States effectively recognized India as a nuclear power through a bilateral nuclear agreement, allowing it to take part in international nuclear trade on the condition that it would continue to support the prevailing nuclear order. According to one set of scholars, this deal was struck to stabilize the non-proliferation regime ‘in the face of challenges to both its legitimacy and effectiveness’ (Frankenbach et al. 2021: 539).

Beyond the co-optation of critics and challengers into existing structures and institutions, legitimacy-seekers frequently attempt to appease critics and neutralize pressures through limited concessions or superficial institutional reforms. For example, several regional global governance institutions have created parliaments or assemblies as a means of pre-empting or deflating democratic critiques (Lenz et al. 2019; see also Grigorescu 2015). An early example from the politics of nuclear armament is the sponsorship by the Truman administration of formal government processes and institutions, including high-profile commissions both at the UN and within the US government, that pre-empted disarmament ideas by ‘accepting the general concerns and language of advocates of international [atomic] control’ while at the same time emphasizing the impossibility of radical action (Meyer 1993: 462). Perhaps the prime example is the 1968 Treaty on the Non-Proliferation of Nuclear Weapons (NPT), through which the established major powers legitimized their own possession of nuclear weapons while at the same time delegitimizing the acquisition of such arms by other states.

Defining the established major powers as ‘nuclear-weapon states’, the NPT ‘effectively legitimizes deterrence’ for a select group of states (Tannenwald 2020: 125; see also e.g. Ritchie 2019: 418; Ruzicka 2018: 372). Here, practices of institutional legitimation work hand-in-glove with discursive practices. After all, institutional legitimation is often amplified through discourses that make use of rules and institutions in rhetorical contests over legitimacy. An example of this is the Russian government’s repeated claims that the NPT gives Russia an internationally recognized ‘right’ to possess nuclear weapons indefinitely: ‘Let us remind those who for some reason wish to close their eyes to this fact that, pursuant to the NPT, five States possess nuclear weapons with absolute legitimacy. All the NPT States parties signed that Treaty and all national Parliaments ratified it’ (Russia 2016). Along similar lines, Nick Ritchie (2013: 157) argues that the British government portrays UK nuclear weapons as objects of ‘institutionally ordained legitimacy’ couched in the NPT. A final example of institutional legitimation in nuclear weapons politics is the entrenchment of nuclear weapons and deterrence in NATO’s institutional identity. In fact, the inclusion in NATO’s 2010 Strategic Concept of the line that NATO is and must remain a ‘nuclear alliance’ came about as a response to the German government’s questioning of the continued deployment of US nuclear weapons on German soil in 2009 (Egeland 2020).

### Behavioural legitimation

A third category of legitimation conceptualized by Bäckstrand and Söderbaum (2018) is ‘behavioural legitimation’. Here, the emphasis is on actions rather than locutions or institutionalization, that is, on practical actions legitimating actors undertake to maintain their licence to operate. As Bäckstrand and Söderbaum point out, institutions often seek to boost their legitimacy by conducting public relations exercises, performance reviews, and opinion polls – in other words through performances geared towards directly or indirectly generating favourable attitudes or associations. A first prominent instantiation of behavioural legitimation in nuclear politics is the provision by legitimating actors of campaign donations: for the last several decades, all successful candidates for US president have accepted significant financial donations from companies that produce nuclear weapon systems (Egeland 2021: 109). In the United States, major defence contractors also routinely pay lobbyists to champion their interests vis-à-vis lawmakers. A more visible example of behavioural legitimation is the North Korean government’s periodic organization of flashy military parades showcasing new arms and missiles. Some analysts also speculated that North Korea in 2018 and 2019 used the Trump–Kim nuclear summits as a vehicle for gaining status and legitimacy as a nuclear power (Moore 2019).

Behavioural legitimation is frequently aimed at building legitimacy ‘by association’, i.e. by building links to something more popular and then leeching on the legitimacy of the latter. This can be achieved through philanthropy, collaborations, sponsorship programmes, the hosting of popular events and spectacles, or by inviting respected people to sit on boards or committees (Dowling and Pfeffer 1975: 133–34; Kobierecki and Strożek 2021). Beyond North Korea’s reported attempts at deriving legitimacy through diplomatic engagement with the United States, an example of the pursuit of behavioural legitimacy ‘by association’ is the UK government’s organizing of a celebratory service at Westminster Abbey in London of 50 years of continuous deployment of British ballistic missile submarines in 2019. The event was not an unambiguous success, however, with some critics arguing that the UK government’s apparent attempt to link nuclear weapons and the Christian faith was not appropriate (e.g. Megoran 2019). As another example, Dima Adamsky (2019) has detailed how the Russian nuclear weapons establishment has encouraged representatives of the Russian Orthodox Church to write and speak in public about the ostensible moral and religious defensibility of Russian nuclear armament. Representatives of the Church have also been invited (or allowed) to sanctify nuclear hardware through religious rituals, involving inter alia the sprinkling of holy water on nuclear missiles and submarines. Through these behaviours, the Russian nuclear complex has been able to develop visible connections with an institution (the Church) many Russians see as a moral authority, helping to allay possible objections to the deployment and potential use of nuclear arms. Adamsky speculates that the acting out of religious rituals in support of the Russian nuclear enterprise could be a means of ensuring that the soldiers in the Russian nuclear chain of command will implement any orders to use nuclear weapons if called upon. The nuclear complex has in other words identified a need to counteract the norm usually referred to as the ‘nuclear taboo’ or ‘tradition of non-use’. Scholars of this norm have detailed how, in the 1950s and 1960s, nuclear weapons came to be seen by many as morally offensive weapons that should not be used under any circumstances (Tannenwald 2008; Paul 2009). Clearly, any such norm conflicts with the credibility of nuclear deterrence postures, meaning that organizations tasked with the exercise of deterrence share an incentive to combat it.

Another salient example of the pursuit of legitimacy ‘by association’ is various defence industrial actors’ sponsorship of the Girl Scouts of America–an organization widely seen as positive and wholesome. Major partners of the Girl Scouts include, for example, nuclear warhead manufacturer Lawrence Livermore and missile companies Raytheon and Lockheed Martin. While these entities’ sponsorship of the Girl Scouts and girls’ education in science, maths, and engineering may of course also be motivated by genuine philanthropic impulses or desires to invest in a future workforce, sponsorship is inextricably entangled in the politics of legitimation, as sponsorship assumes an ‘alignment of values—or at least of interests’ (Frumkin 2006: 272). Consequently, organizations habitually decline offers of gifts and sponsorship if they think the donor is too controversial. For instance, in the 1990s, the tobacco company Philip Morris struggled to find charities that would accept its grant money (Devers et al. 2009: 165). Similarly, some organizations have refused donations from actors involved in the nuclear weapons enterprise, and many banks have in recent years been pressured to divest from corporations involved in the production and maintenance of nuclear arms (Acheson 2018).

As investigated by numerous scholars, actors involved in contested industries frequently attempt to improve or ‘wash’ their image by appearing alongside more popular actors or assuming progressive positions on social issues, be it LGBTQI+ rights (‘pink washing’), feminism (‘purple washing’), or environmental sustainability (‘green washing’). For instance, many companies involved in the nuclear weapons enterprise use social media to brandish and perform liberal, forward-looking values with respect to gender equality and women’s inclusion. As of 15 December 2021, the Twitter account associated with the Boeing Company’s defence industrial activities (@BoeingDefense) had tweeted about ‘women’, ‘girls’, and ‘gender’ more than twice as many times as Boeing’s Twitter accounts associated with civilian aviation (@BoeingAirplanes) and space activities (@BoeingSpace). A similar pattern held also for a number of other defence, space, and aviation companies with multiple Twitter handle, including Raytheon (@RaytheonDefense compared with @RaytheonIntel), Safran (@SafranEngines compared with @SafranElecDef), and Thales (@ThalesDefence compared with @ThalesAerospace).

### Legitimation through information control

Attempts at supressing or amplifying information flows typically fall outside existing typologies of legitimation. But information control provides a frequently-used tool in contests of legitimacy and should therefore be included in the analysis of legitimation practices (see Pelopidas 2022: 187–88). Efforts at controlling information can take the form of ‘withholding information, releasing information at predetermined times, releasing information in juxtaposition with other information that may influence public perception, manufacturing information, communicating information to selective audiences, and distorting information’ (Jowett and O’Donnell 2012: 45–46). Information control thus differs from discursive legitimation insofar as the former works not through persuasion or argumentation but the manipulation of which facts, arguments, and narratives the audience is exposed to in the first place. Direct information control occurs when legitimacy-seekers intervene in immediate and tangible ways to straightforwardly suppress or elevate specific pieces of information, for example through the application of secrecy measures (see Pelopidas 2022; Fraise 2022; Philippe and Statius 2021). Historical examples include the Soviet Union’s efforts at concealing the fates of victims of nuclear testing (Evangelista 1995: 30), the US government’s application of secrecy measures to prevent bad press from the atomic bombings of Hiroshima and Nagasaki (Hook 1988), and the British Broadcasting Corporation’s censoring of the 1965 film *The War Game* (Chapman 2006). A more recent and mundane example highlighted by Joseph Cirincione was the convening in 2021 of a hearing on nuclear strategy in the Strategic Forces Subcommittee in the US Senate. In Cirincione’s (2021) words, the hearing was clearly designed to dispel moral apprehensions about the American nuclear enterprise, with witnesses handpicked to ‘present a nearly uniform endorsement of existing programs and contracts, particularly the controversial new intercontinental ballistic missile.’ The French Ministry of the Armed Forces maintains a policy of inviting select journalists and students to join French ballistic missile submarine patrols as embedded observers (see Guibert 2020). That said, in a context of strong public support for disarmament in most states, those working to sustain the nuclear enterprise will in general have an interest in limiting the political salience of nuclear weapons, that is, to avoid politicization of, and attention to, nuclear affairs in general (see Egeland and Pelopidas 2021: 245).

Information control may also be conducted in more indirect or diffuse ways. For example, governments, organizations, and multinational corporations routinely provide think tanks and research centres with funding for policy research, shaping public debate and the marketplace of ideas (Egeland and Pelopidas 2022; Higgot and Stone 1994). For example, defence contractors, including those involved in the development of nuclear weapon systems, are among the most generous donors to foreign policy think tanks in France, the United Kingdom, and the United States (in China and Russia, the major foreign policy think tanks are closely affiliated with the government). According to William Hartung (2020), weapons makers are eager to both ‘launder’ and amplify their outreach by funding think tanks that can act as outriders with intellectual capital (see also Walt 2009; Raskin 1963). On the flip side, the withholding of funding can be an effective means of muting criticism. For instance, as Simone Turchetti (2021: 545) documents, in the early 1980s NATO administrators ‘agreed to stall the funding of studies on the predicted global catastrophe to be derived from nuclear exchange’ and ‘removed nuclear winter from the list of scientific subjects worth sponsoring to avoid giving greater resonance to this notion.’ At the same time, NATO-sponsored scientists organized meetings in which nuclear winter studies were criticized.

Attempts at controlling information often amount to efforts at building a coherent ideology or ‘hegemony’ that privileges certain perspectives and ways of thinking over others (see Barnett and Duvall 2005). Hegemony-building, in this view, aims at fostering what theorists of organizations have called ‘legitimacy as “taken-for-grantedness”’ (Barron 1998: 207; Suchman 1995: 582). In nuclear weapons politics, this practice is often referred to with more benign terms such as ‘education’ or ‘pedagogy’ (Volpe and Kühn 2017; Guibert 2020; Sayle 2020). Like other fields of knowledge, nuclear expertise is governed by deeply rooted socio-aesthetic norms that promote certain ways of viewing the world and discourage others. In the words of one scholar, there is broad acceptance among experts ‘of what constitutes “serious nuclear scholarship and political theorizing,” and what, conversely, does not.’ Analysts who oppose the basics of nuclear deterrence theory ‘risk decreasing their social and political efficacy in the broader competitive field of nuclear-policy argumentation’ (Meyn 2018: 115; see also Pelopidas 2016). In contrast to straightforward efforts at persuasion, hegemony-building and information control are thus not first and foremost aimed at changing the minds of individual members of an audience but rather at changing conceptions of ‘common sense’ over the long term (Cox 1983; Ritchie 2019). Hegemony-building can be accomplished in several ways, including the promotion of cultural products that amplify favoured narratives. Along these lines, the Pentagon provides input and sponsorship to numerous Hollywood films and series to protect the reputation of the US military (Löfflmann 2013). Scholars have also argued that the museums run by the US government to commemorate and showcase nuclear history encourage a ‘nuclearized’ worldview that privileges the US government’s narratives about the legitimacy of nuclear weapons as instruments of security (Gerster 2013; Pfeiffer 2020). In Russia and China, academic, journalistic, and intellectual freedoms are heavily circumscribed, with criticism of government policy virtually impossible (Xu and Albert 2017; Amnesty International 2022). Table 1 summarizes the four legitimation practices discussed above.

**Table 1:** Typology of legitimation

Type of legitimation	Examples from nuclear politics
Discursive legitimation	Nuclear-armed governments using euphemism, equivocation, and reversification to portray nuclear weapons in a neutral or positive way (‘nukespeak’) Arms contractors promoting reassuring arguments and supportive strategic narratives in traditional and new media
Institutional legitimation	Established major powers freezing the nuclear status quo through the NPT and putative pursuit of multilateral disarmament The Obama administration defining NATO as a ‘nuclear alliance’
Behavioural legitimation	Raytheon, Lockheed Martin, and Lawrence Livermore partnering with the Girl Scouts of America The Russian nuclear complex chasing religious backing through cooperation with the Russian Orthodox Church
Legitimation through information control	The BBC censoring the film *The War Game* Nuclear-armed governments and defence contractors funding think tanks to create a favourable information space

## Conclusion

States, international organizations, and corporations constantly struggle over the legitimacy of particular policies and institutions. In this article, I have argued that the practices of legitimation that make up these struggles could fruitfully be divided into four categories: discursive legitimation, institutional legitimation, behavioural legitimation, and legitimation through information control. These categories capture a broad array of doings and sayings that are frequently on display in national and international politics, including, as I have argued here, the politics of nuclear armament. While some have suggested that legitimation by definition precludes ‘negative’ tactics such as lying or the application of secrecy measures (Hurd 2007: 44), I suggest that all efforts aimed at building or maintaining social license should be analysed within a unified framework. ‘Negative’ legitimation tactics such as information control and co-optation play important roles in shielding controversial social arrangements from normative censure and are thus central to the struggle for legitimation.

Consider, for example, the varied practices the Russian government enacts to legitimise the development, deployment, and potential use of nuclear arms. First, the Russian defence establishment and its outriders in the media engage more or less continuously in discursive practices of legitimation, projecting arguments that frame Russia as a defensively oriented actor obliged to maintain and modernize its nuclear arsenal to protect the Russian people from mighty foes and to counteract humiliations inflicted by the West. Second, Russia benefits from institutional legitimation, perhaps most notably the NPT, which Russian diplomats routinely argue gives Russia a permanent right to possess nuclear weapons. Third, the Russian nuclear complex engages in several behavioural practices of legitimation. For instance, the Russian military has developed informal partnerships with the Russian Orthodox Church to build legitimacy for the Russian nuclear complex through rituals such as the sprinkling of holy water on nuclear missiles. Fourth, the Russian government acts to maintain the legitimacy of its nuclear weapons programme through practices of information control, with critics of the Russian government risking imprisonment and physical harm. In combination, this wide selection of legitimation practices appears to have played an important role in allowing the Russian nuclear complex to thrive in the post-Cold War period.

Disentangling the varied menu of legitimation practices used by political actors is important both to advance scholarly understanding of legitimation and to gauge the prospects for institutional contestation and change. It is also worth reflecting on the fact that the intensity of legitimation efforts appears to be intimately connected, if not strictly proportional, to the intensity of efforts at delegitimation. It seems clear, for example, that the nuclear-armed states that are subject to the most intense disarmament pressures are also the ones most actively engaged in visible efforts at legitimating the existing nuclear order. Legitimating actors’ choice of legitimation strategies thus appears to be conditioned by the wider political context, perhaps most obviously regime type. For instance, the most flagrant information-control tactics used by authoritarian states such as China and Russia are not as readily available (or acceptable) to France, the United States, or the United Kingdom. Clearly, when a pro-nuclear government exercises authoritarian information control, suppressing meaningful civil society debate, there is less of an incentive for nuclear industrial actors to pour money into think tanks and lobby groups to push back against disarmament advocates in public discussions, as is the case in France, India, the United Kingdom, and the United States.

The enactment by nuclear-armed states and companies of the various practices of legitimation discussed in this article strongly suggests that nuclear weapons are vulnerable to campaigns of stigmatization–or at the very least that those currently invested in the nuclear enterprise believe that to be the case. Future research could investigate the relative effectiveness of different types of nuclear and other legitimation practices, including by assessing the reception of various kinds of legitimation by relevant audiences.

## Footnotes

1 The alternative interpretation of the slogan would be that any use of Russia’s strategic nuclear weapons would trigger civilizational collapse and, by extension, ‘silence’. It is not entirely clear, however, why this would be worth celebrating on a Moscow billboard.
